# The complete chloroplast genome of *Utricularia tenuicaulis* Miki (Lentibulariaceae) isolated in Korea

**DOI:** 10.1080/23802359.2022.2080597

**Published:** 2022-06-20

**Authors:** Jongsun Park, Hong Xi, Yongsung Kim, Sang-Tae Kim

**Affiliations:** a InfoBoss Inc., Seoul, Republic of Korea; b InfoBoss Research Center, Seoul, Republic of Korea; c Honam National Institute of Biological Resources, Mokpo, Korea; d Department of Medical & Biological Sciences, The Catholic University of Korea, Bucheon, Republic of Korea

**Keywords:** Chloroplast genome, Lentibulariaceae, phylogenetic analysis, *Utricularia tenuicaulis*

## Abstract

*Utricularia tenuicaulis* Miki 1935 is an aquatic carnivorous plant species found in East Asia including Korea and Japan. In this study, the chloroplast genome of *U. tenuicaulis* was successfully sequenced. The assembled genome (153,976 bp; GC ratio, 37.0%) contains four subregions, with the large single copy (LSC; 84,596 bp; 34.9%) and small single copy (SSC; 17,946 bp; 30.5%) regions separated by 25,718 bp of inverted repeat regions (42.7%), and includes 126 genes (81 protein-coding genes, 8 rRNAs, and 37 tRNAs). Phylogenetic analyses based on the whole-chloroplast genomes of 18 species, including 17 Lentibulariaceae species and one outgroup species, suggest a close relationship between *U. tenuicaulis* and *Utricularia macrorhiza* Leconte 1824. A comparison of genomic variation between *U. tenuicaulis* and *U. macrorhiza* confirmed the validity of the specific discrimination of *U. tenuicaulis.*

Aquatic bladderworts are carnivorous plants containing air-filled floating structures (Miki 1935; Rutishauser 2016). *Utricularia tenuicaulis* Miki 1935 was originally described as an aquatic bladderwort distinguished from *U. japonica* Mak. 1914 by its slenderer scape with a hollow core (Miki 1935; Shin et al. 2006). Taxonomically, *U. japonica* and *U. tenuicaulis* were merged into *U. australis* R. Br. 1810 as the sterile *U. australis* f*. australi*s and the fertile *U. australis* f. *tenuicaulis* by Taylor (1989), following the recommendation of Komiya and Shibata (1980). In East Asia, the distribution of *U. tenuicaulis* closely overlaps those of *U. japonica* (= *U. australi*s f. *australis*) and *U. macrorhiza* (Kadono 1994; Komiya 1997). Following detailed morphological investigation (Kadono 1994; Shin et al. 2006) and experimental confirmation of the origin of sterile *U. australis* through hybridization between *U. tenuicaulis* and *U. macrorhiza* (Kameyama et al. 2005), *U. tenuicaulis* was reconsidered as a distinct species. This taxonomic uncertainty prevented the full evaluation of the phylogenetic position of *U. tenuicaulis* in a recent phylogenetic study (Silva et al. 2018). Therefore, to investigate the taxonomic status of *U. tenuicaulis*, which has been misidentified as *U. japonica* in Korea (Na et al. 2008; Park, An, et al. 2020), we obtained its complete chloroplast genome sequences from a sample collected in Korea.

Total DNA (6.44 µg) was extracted from fresh leaves (600 mg) of *U. tenuicaulis* collected at the Saeteomal wetland at Gunpo-ro, Gyeonggi-do, Korea (37.339967°N, 126.936591°E) using a DNeasy Plant Mini Kit (Qiagen, Hilden, Germany). A voucher specimen was deposited into the InfoBoss Cyber Herbarium (IN; http://herbarium.infoboss.co.kr/; voucher no., IBS-00023; Contact: Jongsun Park; starflr@infoboss.co.kr). Genome sequencing was conducted using a HiSeq4000 system at Macrogen Inc., Korea, and *de novo* assembly was performed using softwares; Velvet v1.2.10 (Zerbino and Birney 2008), GapCloser v1.12 (Zhao et al. 2011), BWA v0.7.17 (Li 2013), and SAMtools v1.9 (Li et al. 2009) in the Genome Information System (GeIS) environment (http://geis.infoboss.co.kr/), which has been used in previous studies (Heo et al. 2020; Lee and Park 2021; Park, Kim, et al. 2021; Park, Min, et al. 2021). The Geneious Prime v2020.2.4 (Biomatters Ltd., Auckland, New Zealand) was used for chloroplast genome annotation based on the *Pinguicula ehlersiae* Speta & Fuchs chloroplast genome (NC_023463).

The *U. tenuicaulis* chloroplast genome (GenBank accession no. MN529625) is 153,976 bp in length, with a GC ratio of 37.0%, and has four subregions; the large single copy (LSC; 84,596 bp; 34.9%) and small single copy (SSC; 17,946 bp; 30.5%) regions separated by two inverted repeats (IRs; 25,718 bp; 42.7%), including 126 genes (81 protein-coding genes, 8 rRNAs, and 37 tRNAs) in the LSC and SSC regions and 17 genes (6 protein-coding genes, 4 rRNAs, and 7 tRNAs) duplicated in the IR regions. We determined four subregions by identifying junctions of two IR regions using the program, ‘BLAST 2 Sequences’ that supports BLAST searches to find the duplicated regions.

We identified 562 single-nucleotide polymorphisms (SNPs) and 302 indel regions (2,201 bp) with the comparison of *U. macrorhiza* (NC_025653). This indicates higher genomic variations in *Utricularia* species comparing to previous reports for other species; 220–520 SNPs and 125-144 indels in *Castanopsis* species (Park, Xi, et al. 2021), 7–27 SNPs and 19–49 indels in the *Viburnum dilatatum* species complex (Park, Xi, et al. 2020).

17 species in Lentibulariacea and *Lippia origanoides* Kunth. 1818 (Verbenaceae) as an outgroup were used for phylogenetic analysis. We used MEGAX (Kumar et al. 2018) to construct maximum-likelihood (ML) and neighbor-joining (NJ) trees and MrBayes v3.2.6 (Ronquist et al. 2012) to perform Bayesian inference (BI) after aligning the full chloroplast genomes using MAFFT v7.450 (Katoh and Standley 2013). We performed a heuristic search using nearest-neighbor interchange branch swapping, the Tamura–Nei model, and uniform rates among sites to construct ML and NJ phylogenetic trees, with default values for other options. To estimate node confidence, we performed bootstrap analyses with 1,000 and 10,000 pseudoreplicates for ML and NJ trees, respectively. For BI analysis, we used the general-time-reversible (GTR) model with gamma rates as the molecular model and a Markov chain Monte Carlo algorithm implemented for 1,100,000 generations. To build the BI consensus tree, we sampled trees every 200 generations after removing 100,000 generations as burn-in. All phylogenetic trees inferred from the ML, NJ, and BI methods showed the same topology, with three genera of Lentibulariaceae grouped with strong support (Figure 1). Our phylogenetic analysis indicated that *U. tenuicaulis* is closely related to but distinguished from *U. macrorhiza* as the phylogenetic distance between two species is similar or larger than those among *Genlisea* species (Figure 1).

**Figure 1.**
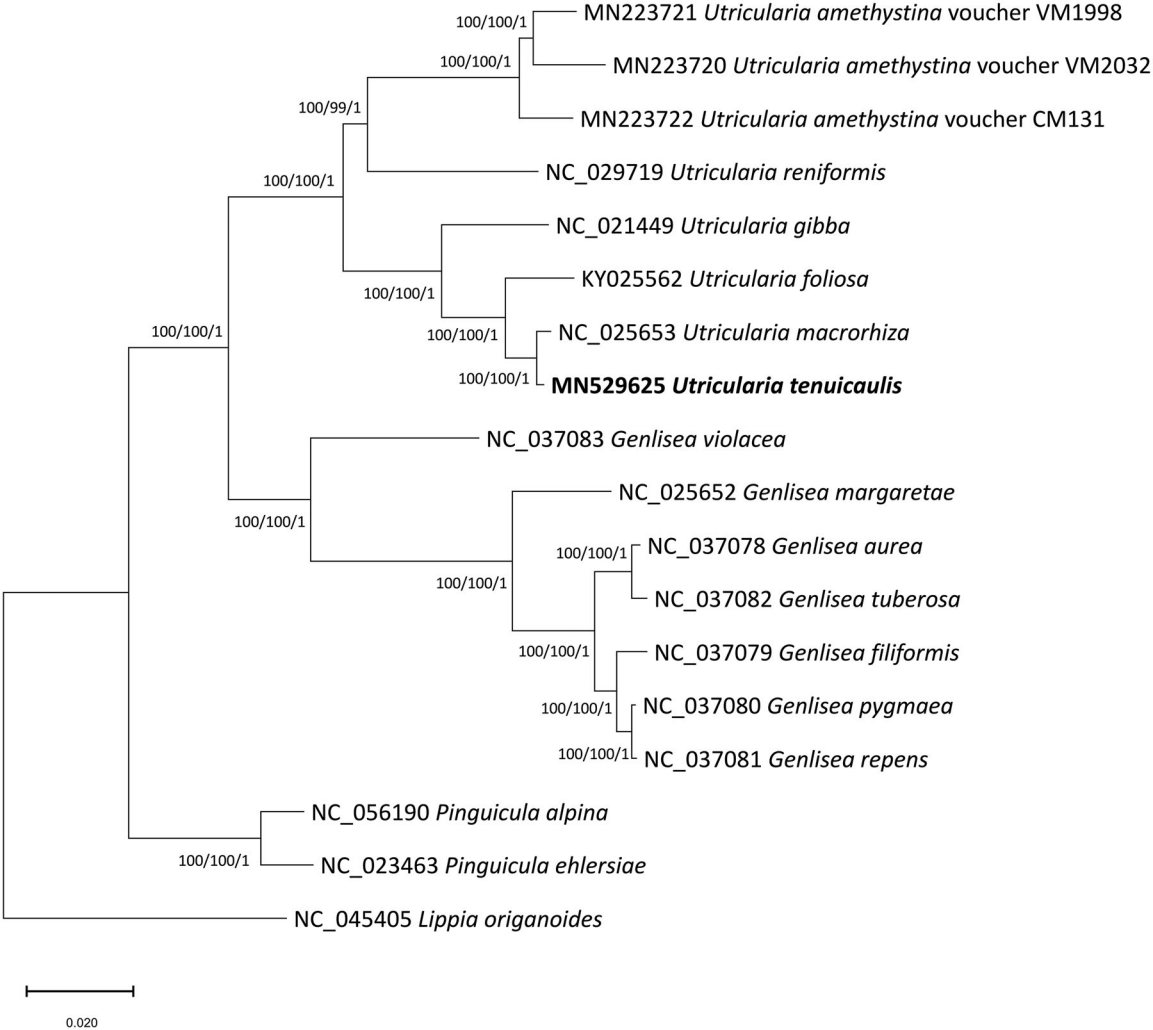
Phylogenetic tree inferred from 18 chloroplast genomes representing 17 Lentibulariaceae species and one outgroup species. The maximum likelihood (ML) tree is presented with bootstrap support values and posterior probabilities estimated using the ML search, neighbor-joining, and Bayesian inference methods.

These results suggest that *U. tenuicaulis* is an independent taxon, genetically distinguished from *U. macrorhiza*. Further sequencing analysis including a wider range of taxa is necessary to clarify the phylogenetic relationships of Utricularian species in greater detail.

## Data Availability

The chloroplast genome sequence data that support the findings of this study are openly available in GenBank of NCBI at (https://www.ncbi.nlm.nih.gov/) under the accession no. MN529625. The associated BioProject, Sequenced Read Archive, and Bio-Sample numbers are PRJNA764600, SAMN21509661, and SRR15970505 respectively.
